# Dysregulation of the MACF1‐Rab14/KIF16B‐FGFR Vesicular Trafficking Axis Skews MSC Lineage Commitment in Glucocorticoid‐Induced Osteoporosis

**DOI:** 10.1002/advs.77073

**Published:** 2026-08-10

**Authors:** Peihong Su, Ye Tian, Fan Zhao, Jing Li, Suryaji Patil, Chunyu Zhu, Yujia Tian, Junrong Wang, Jiating Liu, Aoqi Xiang, Hua Guan, Lusha Zhang, Xiaochang Chen, Airong Qian, Qi Yu

**Affiliations:** ^1^ Shaanxi Provincial Key Laboratory of Ischemic Cardiovascular Disease Institute of Basic and Translational Medicine Xi'an Medical University Xi'an Shaanxi China; ^2^ School of Life Sciences and Technology Northwestern Polytechnical University Xi'an Shaanxi China; ^3^ Department of Radiology & Functional and Molecular Imaging Key Lab of Shaanxi Province Tangdu Hospital Fourth Military Medical University Xi'an Shaanxi China

**Keywords:** FGFR, glucocorticoid‐induced osteoporosis, MACF1, mesenchymal stem cells, vesicle trafficking

## Abstract

Glucocorticoid‐induced osteoporosis (GIO) is driven by a pathogenic shift in bone marrow mesenchymal stem cell (BMSC) lineage commitment from osteogenesis to adipogenesis. Here, we identify Microtubule Actin Cross‐linking Factor 1 (MACF1) as a critical regulator of MSC fate that is severely suppressed during long‐term glucocorticoid (GC) exposure. We demonstrate that MACF1 deficiency phenocopies GC‐induced marrow adiposity and bone loss. Crucially, restoring endogenous MACF1 via promoter‐targeted small activating RNA (saRNA) effectively corrects this fate bias and reverses microarchitectural deterioration in GIO mice. Mechanistically, MACF1 acts as a key facilitator of vesicular trafficking. By orchestrating the assembly of the Rab14/KIF16B complex, MACF1 drives the precise anterograde transport of Fibroblast Growth Factor Receptors (FGFRs) to the plasma membrane. GC exposure downregulates MACF1, disrupting this transport machinery and causing intracellular FGFR sequestration. This transport failure depletes functional cell‐surface FGFRs, thereby blunting osteogenic signaling and triggering the adipogenic shift. Collectively, our findings establish the MACF1‐governed intracellular trafficking network as an important determinant of FGFR spatial distribution and MSC fate, highlighting it as a promising therapeutic target for restoring bone‐fat equilibrium in GIO.

## Graphical abstract

Under normal conditions, MACF1 interacts with Rab14 and facilitates KIF16B‐mediated FGFR vesicle trafficking along microtubules to the plasma membrane, thereby supporting BMSC differentiation. In contrast, chronic GC exposure suppresses MACF1 expression, disrupting this transport and causing intracellular FGFR retention, which blunts osteogenic signaling, skews BMSC fate toward adipogenesis, and ultimately drives glucocorticoid‐induced osteoporosis.

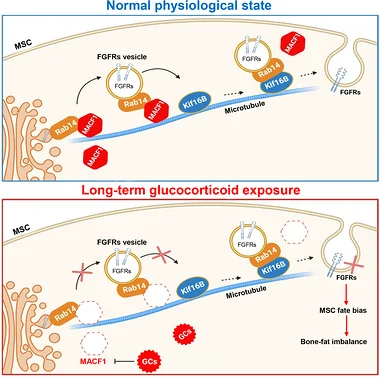

## 1 Introduction

Glucocorticoids (GCs) are widely prescribed for their potent anti‐inflammatory properties, yet prolonged exposure precipitates glucocorticoid‐induced osteoporosis (GIO), the leading cause of secondary osteoporosis and fragility fractures [1]. Dysfunction of multipotent bone marrow mesenchymal stem cells (BMSCs), characterized by an imbalance between osteogenic and adipogenic differentiation, is a key contributor to GIO pathogenesis. Under physiological conditions, BMSCs maintain a “bone‐fat equilibrium” to preserve skeletal homeostasis [2, 3, 4]. This lineage exclusivity is governed by specific transcription factors. Runx2 and Osterix promote osteogenesis, whereas PPARγ and C/EBP family members drive adipogenesis [5, 6]. Besides, the FGF/FGFR signaling pathway functions as an essential molecular rheostat to sustain the bone‐fat equilibrium [7]. For instance, FGF2 binds to FGFR1 and FGFR2, triggering the RAS‐RAF‐MAPK (Erk) cascade and increasing p‐Erk levels. This signaling activation promotes osteogenic differentiation while simultaneously suppressing adipogenesis through PPARγ inhibition [8, 9, 10]. However, long‐term GC exposure dismantles this homeostasis, skewing BMSC lineage commitment toward adipocytes at the expense of osteoblasts [11, 12]. The precise upstream mechanisms by which GCs drive this pathological fate shift remain elusive.Beyond transcriptional programs, the spatiotemporal distribution of receptors has emerged as an equally critical dimension of regulation [13, 14]. The activation of the FGF/FGFR pathway depends on the abundance of FGFRs at the plasma membrane, a feature BMSCs maintain during osteogenesis but gradually lose during adipogenesis [3, 15, 16, 17]. Pharmacological blockade of surface FGFRs suppresses osteogenesis and promotes adipogenesis [18]. Notably, the surface abundance of FGFRs is regulated by their anterograde delivery from the Golgi to the plasma membrane, a process orchestrated by cytoskeletal‐mediated intracellular trafficking [19]. However, whether GCs perturb this trafficking process to compromise surface FGFR levels and thereby skew BMSC fate remains unknown.

Microtubule Actin Cross‐linking Factor 1 (MACF1) functions as a versatile cytoskeletal linker that orchestrates intracellular trafficking and signaling cascades [20]. For example, within the Wnt/β‐catenin signaling pathway, MACF1 facilitates the assembly and transport of the APC complex to promote downstream signaling activation [21]. Beyond its roles in canonical signaling, MACF1 binds specific transcription factors (such as TCF12 and E2F6) to mediate their nuclear translocation [22]. Most relevant to this study, MACF1 has been shown to promote vesicular trafficking by tethering to the Golgi apparatus via its plakin domain and engaging microtubules through its C‐terminal region [23, 24]. Given that FGFR surface abundance is regulated by anterograde vesicular trafficking, and that such trafficking relies on a Rab‐motor complex (Rab14/KIF16B) [25], it is plausible that MACF1 may influence FGFR trafficking by modulating this complex. However, whether MACF1 directly orchestrates this process remains to be elucidated.

In this study, we identify the MACF1‐mediated FGFR vesicular trafficking network as a critical determinant of MSC fate in GIO. Mechanistically, we reveal a previously unrecognized function of MACF1 as a key spatial regulator of the FGF signaling axis. By acting as a scaffold for Rab14/KIF16B complex assembly, MACF1 promotes the anterograde vesicular transport of FGFRs to the plasma membrane. Under long‐term GC exposure, MACF1 downregulation disrupts this transport, causing intracellular FGFR stalling and subsequent attenuation of osteogenic signaling, which drives a pathological adipogenic fate shift. Crucially, reactivating endogenous MACF1 via a bone‐targeted saRNA strategy restores this trafficking efficiency and ameliorates microarchitectural deterioration. Collectively, these findings establish the MACF1‐mediated FGFR transport axis as a major pathological factor of GC‐induced bone loss and highlight its potential as a highly promising therapeutic target.

## 2 Results

### 2.1 Glucocorticoids Drive Bone‐Fat Imbalance by Suppressing MACF1 Expression

To investigate the impact of GCs on bone homeostasis, male C57BL/6 mice were treated with Dex at either a low dose (1 mg/kg) or a high dose (5 mg/kg) three times weekly for 8 weeks. Primary BMSCs isolated from GIO mice exhibited an adipogenic bias accompanied by reduced osteogenesis, characterized by significantly decreased osteogenic markers (*Runx2, Alp*) and mineralization, alongside increased adipogenic markers (*Pparγ, Fabp4*) and lipid accumulation (Figure S1A–E). To elucidate the molecular mechanisms governing this lineage shift, unbiased transcriptomic profiling of primary BMSCs from GIO mice was performed. RNA sequencing identified broad transcriptional alterations, with 1,061 and 943 differentially expressed genes (DEGs) in the low‐ and high‐dose groups, respectively (Figure S2A,B). DEGs were clustered in pathways regulating BMSC fate determination (such as *Runx2, Cebpα*, and *Fabp4*) and intracellular transport (such as *Macf1* and *Vamp7*) (Figure 1A). These transcriptional changes were corroborated by RT‐qPCR in both primary BMSCs derived from GIO mice (Figure 1B) and Dex‐treated C3H10T1/2 cell lines (Figure S2C). Furthermore, pathway enrichment analysis indicated alterations in actin cytoskeleton regulation, the synaptic vesicle cycle, and nucleocytoplasmic transport (Figure 1C), implying that the GC‐induced MSC fate shift is associated with altered intracellular trafficking.

**FIGURE 1:**
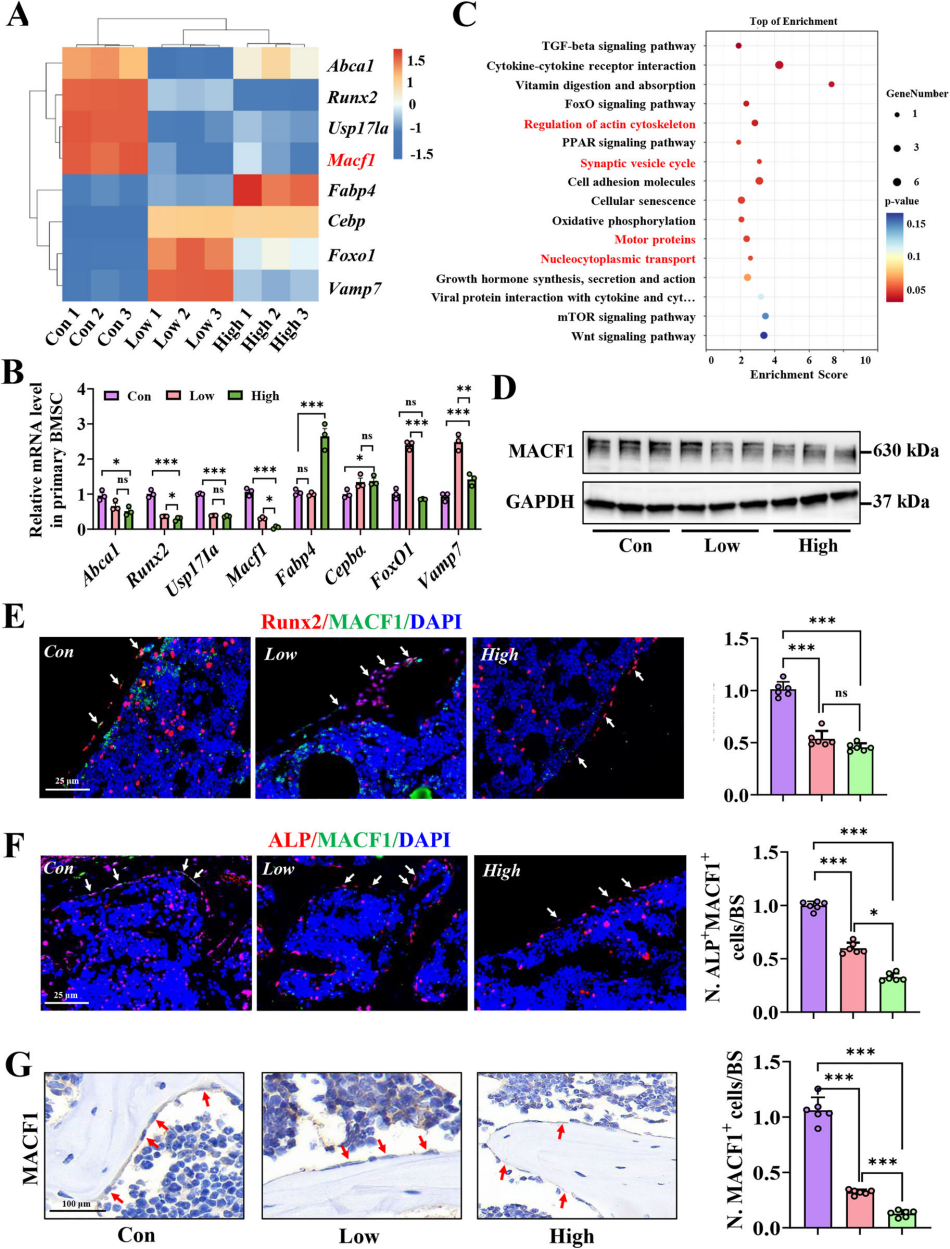
GC suppresses MACF1, thereby disrupting the bone–fat balance. (A) Heatmap representing the DEGs in primary BMSCs isolated from bone tissues of GIO mice, *n* = 3 in each group. (B) Validation of selected DEGs by RT‐qPCR. Gene expression levels were normalized to *Gapdh* and analyzed using the comparative threshold cycle (2^−ΔΔCT^) method, *n* = 3 in each group. (C) The top 20 enriched KEGG pathways among DEGs. The rich factor represents the ratio of DEGs annotated in a pathway to all genes associated with that pathway. (D) MACF1 protein expression level in primary BMSCs isolated from bone tissues of GIO mice, *n* = 3 in each group. (E) Representative immunofluorescence (IF) staining images of MACF1 in Runx2‐positive (Runx2^+^) and (F) ALP‐positive (ALP^+^) cells on the bone formation surface of tibiae and quantitative analysis of the number of Runx2^+^MACF1^+^ cells (N. Runx2^+^MACF1^+^ cells/ BS) and ALP^+^MACF1^+^ cells (N. ALP^+^MACF1^+^ cells/ BS), *n* = 6 in each group. Scale bar, 25 µm. (G) Immunohistochemical (IHC) staining of MACF1 on bone formation surface of tibiae of GIO mice and quantitative analysis of the number of MACF1‐positive cells (N. MACF1^+^ cells/ BS), *n* = 6 in each group. Scale bar, 100 µm. ^*^ *p*<0.05, ^**^ *p*<0.01, ^***^ *p*<0.001.

Given the established role of MACF1 in coordinating cytoskeletal dynamics and transport, we examined its status in the context of GC exposure. Western blot analysis confirmed a downregulation of MACF1 protein in both BMSCs derived from GIO mice (Figure 1D and FigureS2D) and Dex‐treated C3H10T1/2 cell lines (Figure S2E,F). In situ validation via immunofluorescence (IF) staining revealed a reduction of MACF1 in Runx2‐positive (Runx2^+^) and ALP‐positive (ALP^+^) osteolineage cells on the tibial bone formation surface of GIO mice. Interestingly, while the reduction in Runx2^+^MACF1^+^ cells (N. Runx2^+^MACF1^+^ cells/BS) was comparable between dosage groups, the number of ALP^+^MACF1^+^ cells (N. ALP^+^MACF1^+^ cells/BS) showed a greater decrease in the high‐dose group (Figure 1E,F). Immunohistochemistry (IHC) further confirmed the loss of MACF1 on the bone formation surface (Figure 1G). To elucidate the upstream regulation of this MACF1 suppression, an Actinomycin D (Act D) clearance assay was used to evaluate *Macf1* mRNA stability. The results demonstrated that mRNA stability was not altered by Dex treatment, with comparable half‐lives exhibited by both groups (Figure S2G). Collectively, these data suggest that the GC‐induced MSC fate shift might be attributable to downregulated MACF1 levels and altered intracellular trafficking.

### 2.2 MACF1 Maintains the Bone‐Fat Balance by Governing MSC Fate Decisions

To investigate the contribution of MACF1 to MSC lineage commitment, bone‐fat equilibrium was examined in BMSC‐specific MACF1 knockout (MACF1‐cKO) mice. Histological analysis revealed an accumulation of lipid vacuoles within the tibial marrow cavity of MACF1‐cKO mice compared to littermate controls (MACF1‐flox) (Figure 2A). IHC staining further confirmed an upregulation of adipogenic markers including PPARγ and FABP4 (Figure 2B). These data indicate that the loss of MACF1 leads to marrow adiposity and alters bone‐fat homeostasis.

**FIGURE 2:**
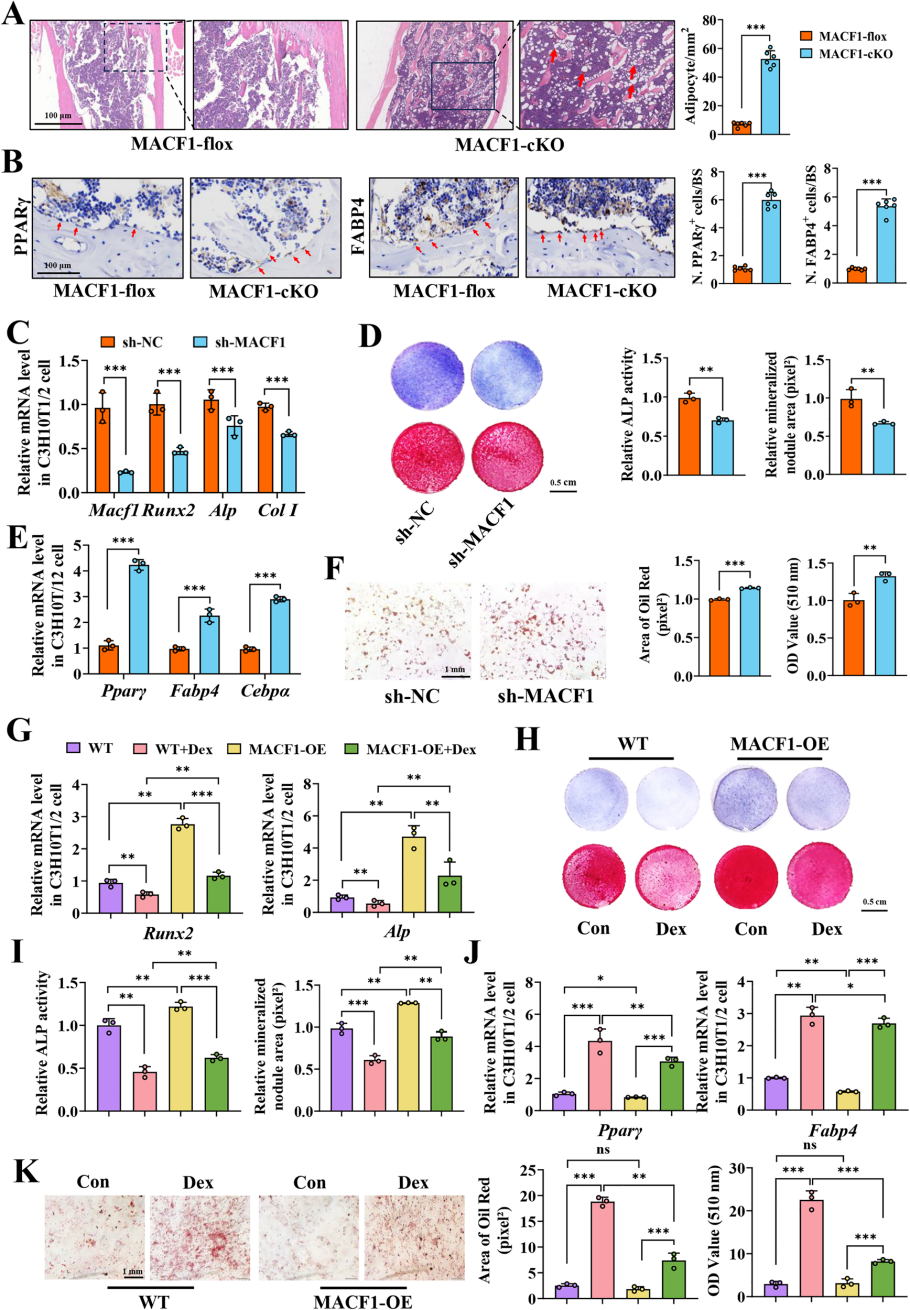
MACF1 promotes osteogenic differentiation and inhibits adipogenesis of MSCs in vitro and in vivo. (A) Representative H&E staining images of the femur of MACF1‐cKO mice, and quantitative analysis of adipocytes in bone marrow, *n* = 6 in each group. Scale bar, 100 µm. (B) Representative IHC staining images of PPARγ and FABP4 on the bone formation surface of femur of MACF1‐flox and MACF1‐cKO mice and quantitative analysis of the number of Pparγ‐positive cells (N. Pparγ^+^ cells/ BS) or FABP4‐positive cells (N. FABP4^+^ cells/ BS), *n* = 6 in each group. Scale bar, 100 µm. (C) The mRNA expression levels of *Macf1* and osteogenic differentiation marker genes in sh‐NC and sh‐MACF1 cells, *n* = 3 in each group. (D) Representative ALP staining images (upper panel) and Alizarin Red S staining images (lower panel) of sh‐NC and sh‐MACF1 cells, and quantitative analysis of ALP activity and area of mineralized nodules, *n* = 3 in each group. Scale bar, 0.5 cm. (E) The mRNA expression levels of adipogenic differentiation marker genes in sh‐NC and sh‐MACF1 cells, *n* = 3 in each group. (F) Representative Oil Red O staining images of sh‐NC and sh‐MACF1 cells and quantitative analysis, *n* = 3 in each group. Scale bar, 1 mm. (G) The mRNA expression levels of osteogenic differentiation marker genes in WT and MACF1‐OE cells treated with Dex, *n* = 3 in each group. (H) Representative ALP staining images (upper panel) and Alizarin Red S staining images (lower panel) in WT and MACF1‐OE cells treated with Dex, and (I) quantitative analysis of ALP activity and area of mineralized nodule, *n* = 3 in each group. Scale bar, 0.5 cm. (J) The mRNA expression levels of adipogenic differentiation marker genes in WT and MACF1‐OE cells treated with Dex, *n* = 3 in each group. (K) Representative Oil Red O staining images of WT and MACF1‐OE cells treated with Dex, and quantitative analysis, *n* = 3 in each group. Scale bar, 1 mm. ^*^ *p*<0.05, ^**^ *p*<0.01, ^***^ *p*<0.001.

Furthermore, MACF1‐knockdown and ‐overexpressing C3H10T1/2 cells were established, and their lineage commitment was subsequently evaluated. Mirroring the effects of GCs, MACF1‐deficient MSCs underwent a fate shift, as evidenced by suppressed osteogenesis with the downregulation of osteogenic markers (*Runx2*, *Alp*, and *Col I*) and decreased ALP activity and mineralization (Figure 2C,D). Conversely, this shift toward an adipogenic lineage was marked by the significant upregulation of adipogenic genes (*Pparγ*, *Fabp4*, and *Cebpα*) and increased lipid droplet accumulation (Figure 2E,F).

However, the lineage fate shift induced by Dex was mitigated by MACF1 overexpression. Specifically, while Dex treatment in wild‐type (WT) cells suppressed osteogenesis and promoted adipogenesis, these effects were reversed in MACF1‐overexpressed (MACF1‐OE) cells. Even in the presence of Dex, osteogenic gene expression, ALP activity, and mineralized nodule formation were maintained at higher levels in MACF1‐OE cells compared to WT cells (Figure 2G–I). Furthermore, the Dex‐induced adipogenic lineage, as shown by marker expression and lipid accumulation, was markedly reduced in MACF1‐OE cells (Figure 2J,K). Collectively, these findings demonstrate that MACF1 downregulation mediates GC‐induced MSC fate shifts, contributing to bone‐fat imbalance.

### 2.3 MACF1 Activation by saRNA Alleviates MSC Fate Bias and Rescues Bone Loss Induced by GCs

To further validate whether restoring endogenous MACF1 expression could rescue the GC‐induced MSC fate bias and subsequent bone loss, a bone‐targeted small activating RNA (saRNA) strategy was utilized. Four saRNAs targeting the *Macf1* promoter (saM1‐4) were designed (Figure S3A) and screened in C3H10T1/2 cells. Among them, saM3 elicited the most potent upregulation of MACF1 at both mRNA and protein levels relative to the negative control (saNC) (Figure S3B–D). Functionally, basal osteogenic differentiation was enhanced by saM3, as evidenced by significantly upregulated marker genes, increased ALP activity, and mineralized nodule formation (Figure S3E–G).

Next, the efficacy of saM3 in counteracting the GC‐induced BMSC fate bias was evaluated in vitro. Primary BMSCs were treated with Dex following transfection with saM3. RT‐qPCR and Western blot analyses confirmed that MACF1 levels and osteogenic gene expression in saM3‐transfected cells were upregulated compared with saNC‐transfected cells, under Dex exposure (Figure 3A,B and Figure S3H). Furthermore, ALP activity and mineralized nodule formation in saM3‐transfected cells were greater than those in saNC‐transfected cells after treatment with Dex (Figure 3C,D). Conversely, lipid formation was significantly decreased in saM3‐transfected cells compared with that in saNC‐transfected cells after treatment with Dex (Figure 3E). These findings demonstrate that targeted activation of MACF1 via saRNA effectively reverses the GC‐induced BMSC fate bias.

**FIGURE 3:**
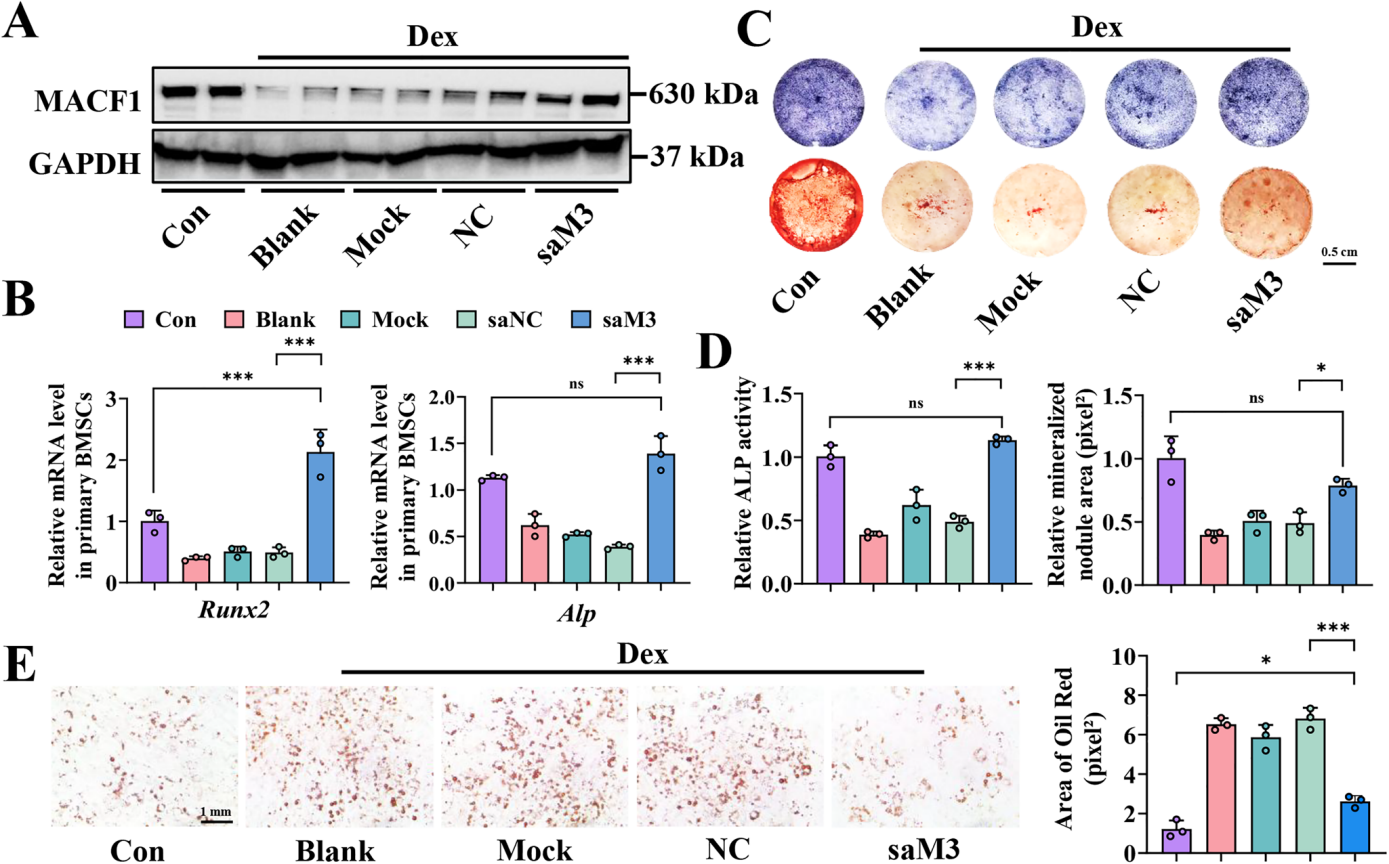
saRNA‐mediated MACF1 activation rescues GC‐induced BMSC fate bias. (A) MACF1 protein level in primary BMSCs transfected with saM3 after treatment with Dex. (B) Expression levels of osteogenic differentiation marker genes in saM3‐transfected primary BMSCs after Dex treatment, *n* = 3 in each group. (C) Representative ALP staining images (upper panel) and Alizarin Red S staining images (lower panel) of saM3‐transfected primary BMSCs upon Dex treatment, and (D) quantitative analysis of ALP activity and area of mineralized nodule, *n* = 3 in each group. Scale bar, 0.5 cm. (E) Representative Oil Red O staining images of saM3‐transfected primary BMSCs after treatment with Dex, and quantitative analysis, *n* = 3 in each group. Scale bar, 1 mm. ^*^ *p*<0.05, ^**^ *p*<0.01, ^***^ *p*<0.001.

To validate the therapeutic potential of saM3 to GC‐induced bone loss in vivo, we established a GIO mouse model (Dex 2.5 mg/kg) and administered saM3 using a bone‐targeted delivery system (SDSSD‐modified polyvinylamine) [26] via tail vein injection (Figure S4A). The treatment demonstrated a safety profile, showing no significant body weight fluctuations or systemic toxicity (liver/kidney histology) (Figure S4B,C).

Furthermore, the expression of MACF1 and osteogenic markers in the femurs of saM3‐treated GIO mice was confirmed by IHC and RT‐qPCR analyses. The results showed that MACF1, ALP, and Runx2 levels were significantly elevated in saM3‐treated mice compared to saNC‐treated mice, but were comparable to those of the Con group (Figure 4A,B and Figure S4D). Calcein labeling indicated that bone mineral deposition rates (BMR) in the saM3 group were significantly higher compared to those in the saNC group, although they remained lower than those in the Con group (Figure 4C). Micro‐CT analysis revealed that GIO mice treated with saM3 exhibited significantly higher bone mass (bone mineral density, BMD; bone volume fraction, BV/TV) and improved trabecular structure compared to the saNC group, though these parameters remained below those of the Con group (Figure 4D,E). The three‐point bending test showed that femoral strength in the saM3 group was enhanced compared to the saNC group, as evidenced by a significant increase in maximum load (Figure 4F). Collectively, these results demonstrate that saRNA‐mediated activation of MACF1 mitigates GC‐induced osteogenic suppression and partially ameliorates bone loss in vivo.

**FIGURE 4:**
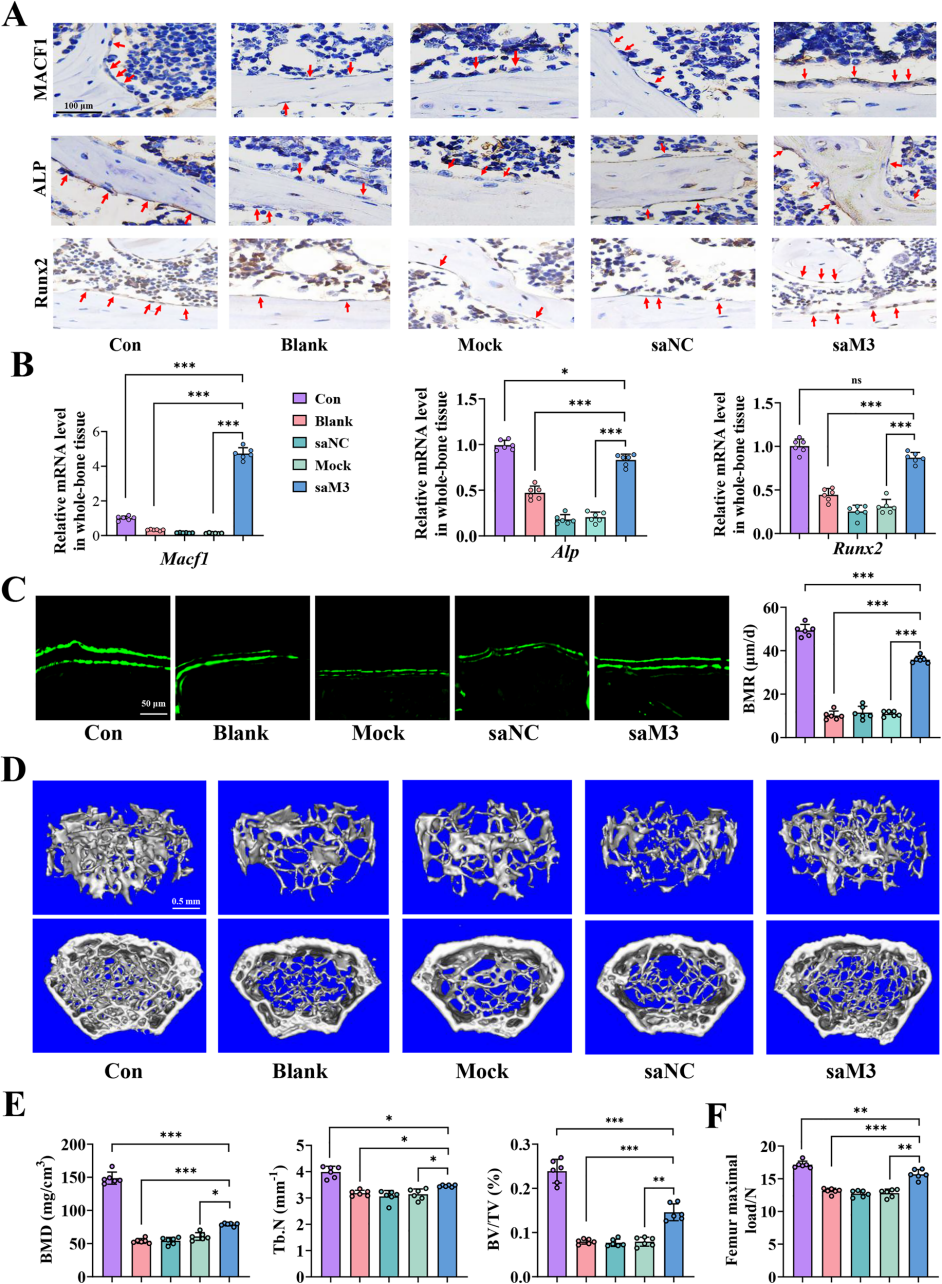
saM3 rescues GC‐induced bone loss. (A) IHC staining of MACF1, Runx2 and ALP in tibia sections from GIO mice treated with or without saM3, *n* = 3 in each group. Scale bar, 100 µm. (B) *Macf1*, *Runx2*, and *Alp* mRNA expression levels in femurs from GIO mice treated with or without saM3, *n* = 6 in each group. (C) Double calcein labeling images of tibias showing new bone formation from GIO mice treated with or without saM3 and quantitative analysis, MAR, mineral apposition rate. *n* = 6 in each group. Scale bar, 50 µm. (D) Representative trabecular bone 3D µ‐CT images from distal femurs of GIO mice treated with or without saM3. Scale bars, 0.5 mm. (E) Quantitative µ‐CT analysis of BMD, BV/TV and Tb.N in GIO mice treated with or without saM3, *n* = 6 in each group. (F) Femur maximal load of GIO mice treated with or without saM3 was determined using three‐point bending, *n* = 6 in each group. ^*^ *p*<0.05, ^**^ *p*<0.01, ^***^ *p*<0.001.

### 2.4 Rab14, a Binding Partner of MACF1, Recruits KIF16B to Facilitate FGFR Vesicle Trafficking

To clarify the mechanism by which long‐term GC exposure induces MSC fate bias through suppressing MACF1, MACF1‐interacting proteins were identified via Co‐IP coupled with LC‐MS/MS using sh‐MACF1 and sh‐NC cells (Tables S1–S4). Subsequent pathway enrichment analysis was performed using the identified interacting proteins. Pathway analysis revealed that several top‐ranked pathways, primarily related to motor proteins, protein export, and the synaptic vesicle cycle, were significantly enriched (Figure 5A). Consistent with prior evidence, this enrichment in transport‐related processes implicates the pivotal role of MACF1 in mediating vesicle trafficking from the Golgi to the periphery [23, 24]. Furthermore, recent findings have identified Rab14 as a recruiter of KIF16B, thereby mediating FGFR vesicle trafficking [25]. In our data, Rab14 was identified as a MACF1‑binding partner in sh‑NC cells, but this interaction was lost (below LOD) in sh‑MACF1 cells (Figure S5A). This interaction was further confirmed by in vivo IF staining, which showed the colocalization of MACF1 and Rab14 within Runx2^+^ cells on the bone formation surface of MACF1‐flox mice; however, this colocalization was absent in MACF1‐cKO mice (Figure 5B). In agreement with these in vivo findings, MACF1 colocalized with Rab14 in sh‐NC cells, while the colocalization was decreased in sh‐MACF1 cells (Figure 5C). To further validate the MACF1‐Rab14 interaction, endogenous reciprocal Co‐IP assays were performed. As shown in Figure 5D,E, Rab14 was successfully precipitated by the MACF1 antibody in sh‐NC cells. However, the amount of co‐precipitated Rab14 was reduced in sh‐MACF1 cells, confirming the specificity of the MACF1‐Rab14 interaction. Reciprocally, MACF1 was also precipitated by the Rab14 antibody in sh‐NC cells, and this interaction was similarly diminished in sh‐MACF1 cells (Figure 5E). To further investigate the role of MACF1 in the assembly of the Rab14/KIF16B complex, the binding of Rab14 with KIF16B was analyzed. Reciprocal Co‐IP analyses revealed that while Rab14 and KIF16B co‐precipitated in sh‐NC cells, their interaction was reduced in sh‐MACF1 cells (Figure 5F). Then, pmCherry‐Rab14 and pEGFP‐KIF16B were co‐transfected into sh‐NC and sh‐MACF1 cells, and the colocalization of mCherry‐Rab14 and GFP‐KIF16B was observed. In sh‐NC cells, both proteins exhibited strong colocalization at peripheral punctate structures resembling vesicles. In contrast, in sh‐MACF1 cells, the colocalization was reduced, accompanied by an aberrant perinuclear accumulation of Rab14 and a diffuse distribution of KIF16B (Figure 5G). These data illustrate that MACF1 acts as a scaffold for Rab14/KIF16B complex formation.

**FIGURE 5:**
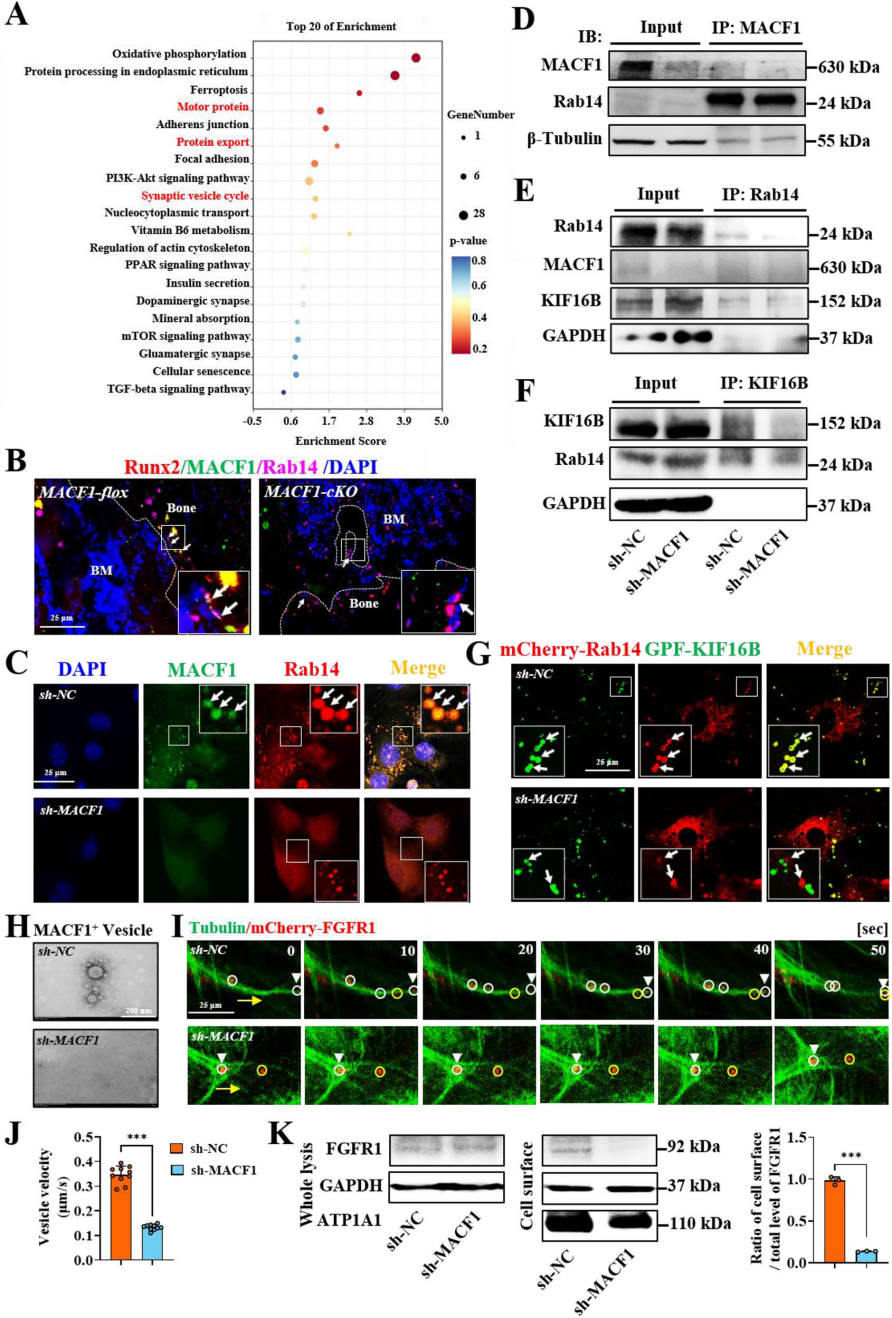
MACF1 mediates Rab14/KIF16B complex formation and promotes FGFR‐containing vesicle trafficking. (A) The top 20 enriched KEGG pathways among MACF1‐interacting proteins identified by Co‐IP. The rich factor represents the ratio of enriched proteins annotated in a pathway to all proteins associated with that pathway. (B) Representative IF staining images of colocalization of MACF1 and Rab14 within Runx2^+^ cells on the bone formation surface of femur from MACF1‐flox and MACF1‐cKO mice, and (C) in sh‐NC and sh‐MACF1 cells. Scale bar, 25 µm. (D–F) Co‐IP analysis of MACF1‐Rab14 and Rab14‐KIF16B interactions in sh‐NC and sh‐MACF1 cells. (G) Representative images of colocalization of Rab14 and KIF16B in sh‐NC and sh‐MACF1 cells co‐transfected with pmCherry‐Rab14 and pEGFP‐KIF16B. Scale bar, 25 µm. (H) Representative scanning electron microscopy (SEM) of MACF1^+^ vesicles in sh‐NC and sh‐MACF1 cells. Scale bar, 200 nm. (I) Real‐time imaging of FGFR1 vesicle trafficking in sh‐NC and sh‐MACF1 cells, captured by spinning‐disc confocal microscopy at 1 frame/10 s after 20 ng/mL FGF2 stimulation and (J) quantitative analysis of vesicle velocity, *n* = 10 in each group. White and yellow circles, FGFR1‐positive vesicles; white arrowhead, an immobile reference point; yellow arrow, direction of vesicle trafficking. Scale bar, 25 µm. (K) Immunoblot analysis of total and membrane FGFR1 levels in sh‐NC and sh‐MACF1 cells (ATP1A1 as membrane marker) and quantitative analysis of the membrane‐to‐total FGFR1 ratio, *n* = 3 in each group. ^*^ *p*<0.05, ^**^ *p*<0.01, ^***^ *p*<0.001.

To verify the role of MACF1 in Rab14/KIF16B‐mediated FGFR vesicle trafficking, intracellular vesicles were immunoisolated via hypotonic lysis followed by anti‐MACF1 immunoprecipitation. SEM analysis confirmed the presence of distinct vesicle‐like structures in sh‐NC lysates, whereas these structures were undetectable in sh‐MACF1 samples (Figure 5H). Further IF staining revealed the colocalization of FGFR1 with MACF1 in sh‐NC cells, accompanied by a diffuse cytoplasmic distribution. In contrast, this colocalization was reduced in sh‐MACF1 cells, where FGFR1 was observed to be sequestered in the perinuclear region with reduced peripheral distribution (Figure S5B). A similar distribution pattern of FGFR2 was also observed in sh‐NC and sh‐MACF1 cells (Figure S5C). Real‐time tracking of mCherry‐FGFR1 revealed that while vesicles in sh‐NC cells underwent active anterograde transport toward the plasma membrane with extensive, long‐range trajectories, those in sh‐MACF1 cells remained largely static with confined movement paths (Figure 5I, Figure S5D and Movies S1 and S2). Consistently, quantitative analyses demonstrated a rapid and continuous increase in the mean squared displacement (MSD) of vesicles in sh‐NC cells, whereas MACF1 deficiency resulted in a flattened MSD curve (Figure S5E). In line with this restricted movement, the average vesicle velocity was markedly reduced upon MACF1 knockdown, from approximately 0.35 µm/s in sh‐NC cells to roughly 0.14 µm/s (Figure 5J). Biochemical fractionation showed that despite elevated total protein levels, the surface‐to‐total ratio of FGFR1 was significantly decreased in sh‐MACF1 cells compared with that in sh‐NC cells, indicating intracellular retention (Figure 5K). The change in the surface abundance of FGFR2 was similar to that of FGFR1 (Figure S5F,G). To assess whether MACF1 deficiency impairs FGF/FGFR signaling, a pathway that regulates MSC fate through the RAS‐RAF‐MAPK (Erk) cascade and elevated p‐Erk1/2 levels, Erk1/2 phosphorylation over a 60‐min time course (0, 5, 10, 30, and 60 min) was detected following FGF2 stimulation (50 ng/mL). Western blot analysis showed that while total Erk1/2 levels remained constant, p‐Erk1/2 levels markedly increased after 5 min of FGF2 treatment in sh‐NC cells and then decreased from 10 to 60 min. In contrast, MACF1 knockdown cells failed to mount this response, exhibiting a progressive decrease in p‐Erk1/2 throughout the 60‐min course (Figure S5H). These results demonstrate that MACF1 deficiency impairs FGF/FGFR signal transduction, providing causal evidence that defective FGFR trafficking leads to attenuated downstream signaling. Functionally, following stimulation with an FGF2 dose gradient, sh‐NC cells exhibited a continuous dose‐dependent increase in ALP activity. Conversely, sh‐MACF1 cells reached a differentiation plateau at 20 ng/mL FGF2 and consistently displayed lower ALP activity than sh‐NC cells (Figure S5I). These results demonstrate that MACF1 deficiency inhibits FGFR vesicle trafficking by impairing Rab14/KIF16B complex formation.

### 2.5 GCs Impair the Rab14/KIF16B Complex Formation and Thereby Inhibite FGFR Vesicle Trafficking Through Down‐Regulating MACF1

To determine whether GC‐induced MSC fate bias resulted from defective Rab14/KIF16B‐mediated FGFR vesicle trafficking, the MACF1/Rab14 interaction in Dex‐treated C3H10T1/2 cells was first examined. IF staining showed that MACF1 colocalized with Rab14 in Con cells, while this colocalization was reduced in Dex‐treated cells (Figure 6A). To visualize the assembly of the Rab14/KIF16B complex in situ, cells were co‐transfected with pmCherry‐Rab14 and pEGFP‐KIF16B followed by Dex treatment. In Con cells, Rab14‐labeled vesicles were uniformly distributed and colocalized with KIF16B, particularly at the cell periphery. Conversely, Dex treatment induced the formation of aberrant, irregular Rab14 clusters sequestered in the perinuclear region, accompanied by a decrease in cytoplasmic distribution (Figure 6B). Co‐IP assays further revealed that Dex compromised the binding of MACF1 to Rab14, thereby attenuating the subsequent recruitment of KIF16B (Figure 6C). Collectively, these data suggest that GC‐induced MACF1 suppression dismantles the Rab14/KIF16B complex.

**FIGURE 6:**
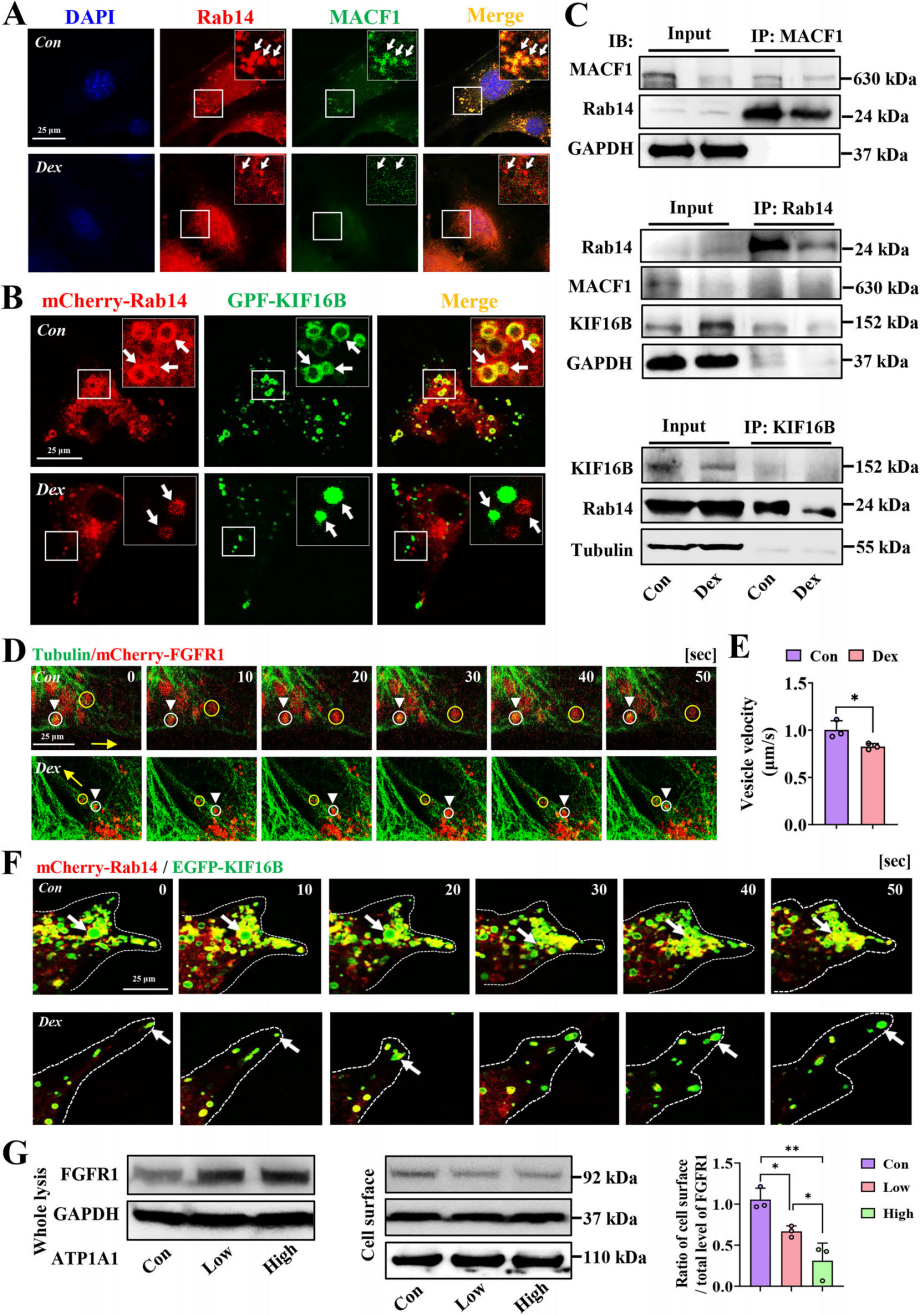
GCs disrupt Rab14/KIF16B‐mediated FGFR‐containing vesicle trafficking by downregulating MACF1. (A) Representative IF staining images of the colocalization of MACF1 (green) and Rab14 (red) in C3H10T1/2 cells treated with Dex. Scale bar, 25 µm. (B) Representative images of the colocalization of Rab14 and KIF16B in C3H10T1/2 cells co‐transfected with pmCherry‐Rab14 and pEGFP‐KIF16B following treatment with Dex. Scale bar, 25 µm. (C) Co‐IP analysis of MACF1‐Rab14 and Rab14‐KIF16B interactions in C3H10T1/2 cells treated with Dex. (D) Real‐time imaging of FGFR1 vesicle trafficking in C3H10T1/2 cells treated with Dex, captured by spinning‐disc confocal microscopy at 1 frame/10 s after 20 ng/mL FGF2 stimulation and quantitative analysis of vesicle velocity. White and yellow circles, FGFR1‐positive vesicles; white arrowhead, an immobile reference point; yellow arrow, direction of vesicle trafficking. Scale bar, 25 µm. (E) Real‐time imaging of Rab14‐KIF16B‐labeled vesicles in C3H10T1/2 cells treated with Dex, captured by spinning‐disc confocal microscopy at 1 frame/10 s after 20 ng/mL FGF2 stimulation. White dashed line, cell boundary. Scale bar, 25 µm. (F) Immunoblot analysis of total and membrane FGFR1 levels in C3H10T1/2 cells treated with Dex and quantitative analysis of the membrane‐to‐total FGFR1 ratio, *n* = 3 in each group. ^*^ *p*<0.05, ^**^ *p*<0.01.

Subsequently, Rab14/KIF16B‐mediated FGFR vesicle trafficking in Dex‐treated C3H10T1/2 cells was assessed. IF analysis revealed that in Con cells, both FGFR1 and FGFR2 were distributed at the cell periphery and exhibited colocalization with MACF1. However, Dex treatment induced a dose‐dependent reduction of FGFR1 and FGFR2 at the cell periphery, accompanied by their sequestration in the perinuclear region and a decrease in colocalization with MACF1 (Figure S6A,B). Similarly, the distribution of FGFR1 in human mesenchymal stem cells (hMSCs) treated with Dex was comparable to that in Dex‐treated C3H10T1/2 cells (Figure S6C). Live‐cell confocal imaging revealed active anterograde transport of FGFR1 vesicles toward the plasma membrane with extensive, long‐range trajectories in Con cells; conversely, Dex treatment caused these vesicles to stall in the perinuclear region with confined movement paths (Figure 6D, Figure S6D and Movies S3 and S4). In agreement with these observations, quantitative analyses demonstrated a rapid and continuous increase in the mean squared displacement (MSD) of vesicles in Con cells, whereas Dex treatment led to a flattened MSD curve (Figure S6E). Consistent with this restricted movement, the average vesicle velocity was significantly reduced from approximately 1.0 µm/s in Con cells to roughly 0.85 µm/s following Dex treatment (Figure 6E). Live tracking further revealed that Dex impaired Rab14/KIF16B complex formation, reducing the movement of Rab14^+^KIF16B^+^ vesicles to the cell membrane compared with untreated cells (Figure 6F and Movies S5 and S6). Subsequently, biochemical fractionation showed that despite an increase in total FGFR1 and FGFR2 levels, their surface abundance and cell surface‐to‐total ratios were significantly reduced in a dose‐dependent manner (Figure 6G and Figure S6F,G). For FGF/FGFR pathway, western blot analysis showed that while total Erk1/2 levels remained constant, p‐Erk1/2 levels in Con cells clearly increased at 5 min of FGF2 treatment before declining from 10 to 60 min. Conversely, despite exhibiting higher basal p‐Erk1/2 levels than Con cells, Dex‐treated cells showed a progressive decrease in phosphorylation throughout the 60 min course (Figure S6H), indicating suppressed FGF/FGFR signaling. Functionally, following stimulation with an FGF2 dose gradient, Con cells exhibited a continuous, dose‐dependent increase in ALP activity and mineralization. However, Dex‐treated cells displayed a blunted osteogenic response to FGF2 stimulation, with ALP activity and mineralization plateauing at lower levels (Figure S6I,J). These findings demonstrate that by suppressing MACF1, the regulator of FGFR vesicular trafficking, GCs attenuate the Rab14/KIF16B‐mediated delivery of FGFRs to the membrane, thereby contributing to MSC fate bias.

### 2.6 MACF1 Overexpression Rescues GC‐Induced MSC Fate Shift by Restoring Rab14/KIF16B‐Mediated FGFR Vesicle Trafficking

To further validate the role of MACF1 in Rab14/KIF16B‐mediated FGFR vesicular trafficking and subsequent MSC fate determination, the interactions between MACF1 and Rab14, as well as Rab14 and KIF16B, were assessed in WT and MACF1‐OE cells. IF staining and Co‐IP assays confirmed that, compared with WT cells, MACF1 overexpression enhanced the MACF1‐Rab14 interaction, thereby promoting Rab14/KIF16B complex formation (Figure 7A–C). Furthermore, FGFR membrane abundance was evaluated in Dex‐treated WT and MACF1‐OE cells. Although Dex significantly suppressed FGFR1 membrane localization while increasing its total protein levels, MACF1 overexpression mitigated this effect, as evidenced by an elevated cell surface‐to‐total FGFR1 ratio (Figure 7D,E). These results indicate that MACF1 overexpression restored FGFR vesicular trafficking under Dex exposure by promoting Rab14/KIF16B complex assembly.

**FIGURE 7:**
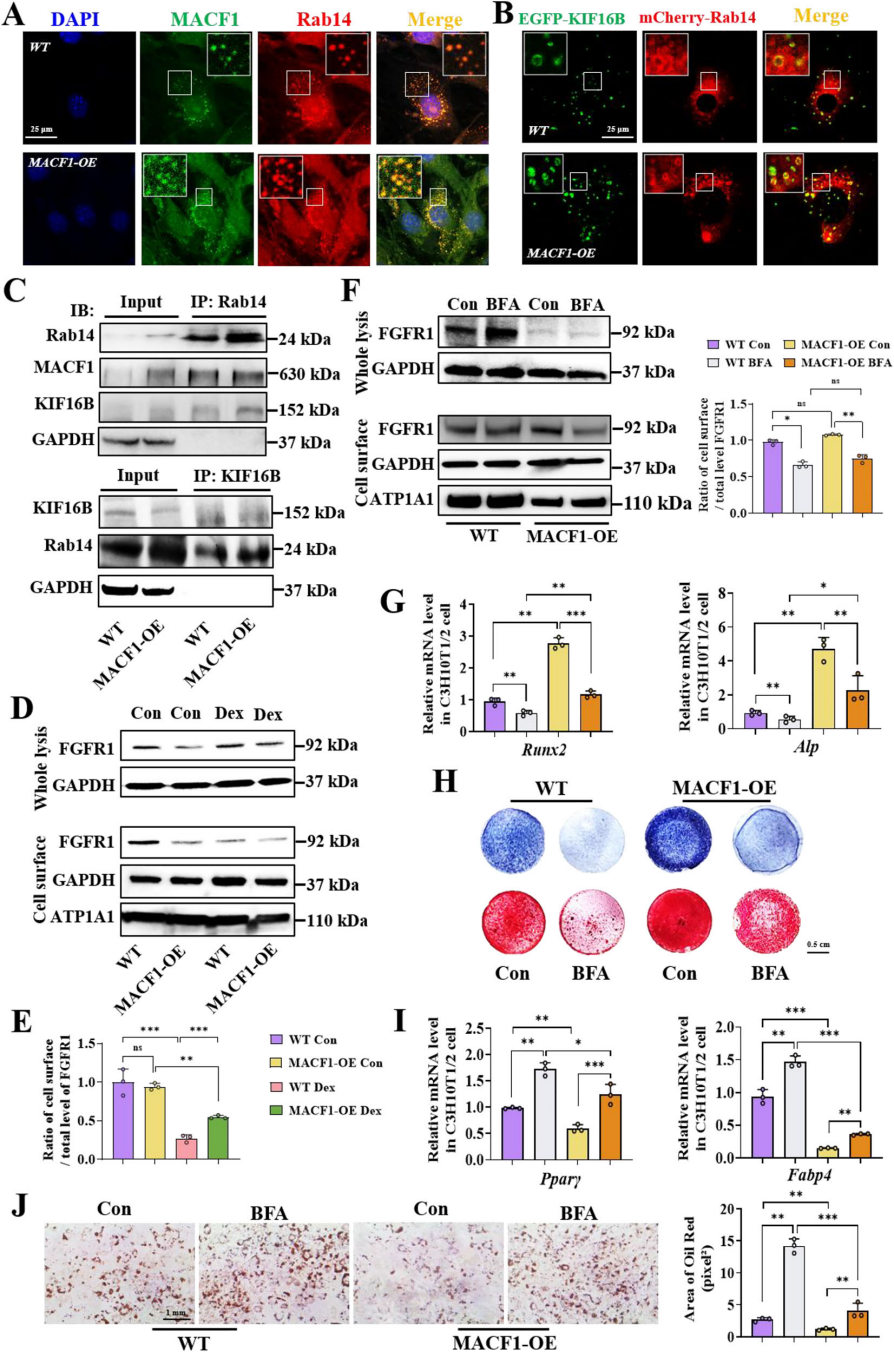
MACF1 overexpression enhances Rab14/KIF16B‐mediated FGFR‐vesicle trafficking to reverse the GC‐induced MSC fate shift. (A) Representative IF staining images of the colocalization of MACF1 (green) and Rab14 (red) in WT and MACF1‐OE cells. Scale bar, 25 µm. (B) Representative images of the colocalization of Rab14 (mCherry) and KIF16B (EGFP) in WT and MACF1‐OE cells co‐transfected with pmCherry‐Rab14 and pEGFP‐KIF16B. Scale bar, 25 µm. (C) Co‐IP analysis of MACF1‐Rab14 and Rab14‐KIF16B interactions in WT and MACF1‐OE cells. (D) Immunoblot analysis of total and membrane FGFR1 levels in WT and MACF1‐OE cells treated with Dex and quantitative analysis of the membrane‐to‐total FGFR1 ratio, *n* = 3 in each group. (E) Immunoblot analysis of total and membrane abundance of FGFR1 in WT and MACF1‐OE cells treated with BFA and (F) quantitative analysis of the membrane ‐to‐total FGFR1 ratio, *n* = 3 in each group. (G) *Runx2* and *Alp* mRNA expression level in WT and MACF1‐OE cells treated with BFA, *n* = 3 in each group. (H) Representative ALP staining images (upper panel) and Alizarin Red S staining images (lower panel) of WT and MACF1‐OE cells treated with BFA. Scale bar, 0.5 cm. (I) *Pparγ* and *Fabp4* mRNA expression levels in WT and MACF1‐OE cells treated with BFA, *n* = 3 in each group. (J) Representative Oil red O staining of WT and MACF1‐OE cells treated with BFA and quantitative analysis of the area of Oil Red, *n* = 3 in each group. Scale bar, 1 mm. ^*^ *p*<0.05, ^**^ *p*<0.01, ^***^ *p*<0.001.

To further elucidate the role of this vesicle trafficking in MSC fate determination, C3H10T1/2 cells were treated with Brefeldin A (BFA), a specific vesicle transport inhibitor. Initial CCK‐8 assays identified 5 µM as an optimal concentration that effectively blocked transport without compromising cell viability (Figure S7A). Similar to GC treatment or MACF1 deficiency, BFA reduced the surface abundance and the cell surface‐to‐total ratio of both FGFR1 and FGFR2, despite an increase in their total protein levels (Figure S7B). Consequently, this trafficking defect suppressed osteogenic differentiation, which was confirmed by the downregulation of osteogenic markers (*Runx2* and *Alp*), decreased ALP activity, and reduced matrix mineralization (Figure S7C–E). Furthermore, BFA pretreatment exacerbated the Dex‐induced inhibition of osteogenesis, leading to a further decline in osteogenic gene expression, ALP activity, and mineralized nodule formation (Figure S7F–H). To determine whether MACF1 could attenuate this trafficking blockade, WT and MACF1‐OE cells were subsequently treated with 5 µM BFA. While BFA broadly reduced the surface abundance of FGFR1/2 in a Dex‐like manner, MACF1‐OE cells maintained higher membrane FGFR levels compared with their WT counterparts (Figure 7F and Figure S7I). Functionally, although BFA exposure suppressed osteogenesis and promoted adipogenesis in both cell types, MACF1 overexpression conferred a significant protective effect. This was evidenced by superior osteogenic capacity (higher ALP activity and mineralization) and notably reduced lipid droplet formation in MACF1‐OE cells relative to WT cells under BFA stress (Figure 7G–J and Figure S7J). Collectively, these findings demonstrate that MACF1 overexpression effectively corrects the GC‐induced MSC fate shift by promoting Rab14/KIF16B‐mediated FGFR vesicular trafficking.

In addition, a Rab14‐knockout (R‐KO) C3H10T1/2 cell line was generated (Figure S8A) to investigate the specific role of Rab14 in GC‐induced MSC lineage commitment. IF staining revealed that FGFR1 was distributed throughout the cytoplasm in R‐KO cells, with reduced membrane‐associated signal, compared with control (R‐NC) cells; this distribution pattern remained unaltered following Dex treatment (Figure S8B). Functionally, both osteogenic and adipogenic capacities were reduced in R‐KO cells compared with R‐NC cells, and these basal phenotypes remained unaffected by subsequent Dex exposure (Figure S8C–H). These data indicate that Rab14 knockout renders MSCs insensitive to Dex treatment, suggesting that Rab14 functions as an essential downstream effector of the MACF1‐Rab14/KIF16B‐FGFR trafficking axis.

## 3 Discussion

In this study, we identify MACF1 as an essential spatial regulator of BMSC fate determination in GIO, dictating this process by orchestrating the Rab14/KIF16B‐mediated vesicular trafficking of FGFRs. Under physiological conditions, MACF1 acts as an indispensable scaffold that ensures receptor transport to the plasma membrane. However, long‐term GC exposure suppresses its expression, dismantling this transport machinery. This GC‐induced deficiency paralyzes FGFR delivery and causes intracellular sequestration. Consequently, this spatial trafficking failure blunts vital FGF signaling, driving a pathogenic lineage shift from osteogenesis to adipogenesis. Collectively, our findings establish that the collapse of MACF1‐governed vesicular trafficking dictates MSC fate bias, providing novel insights into GIO etiology and highlighting this trafficking axis as a promising therapeutic target.

BMSCs maintain skeletal homeostasis through a delicate bone‐fat balance, which is regulated by several essential transcription factors and signaling pathways. Runx2 and Osterix drive osteogenesis, whereas PPARγ and C/EBPα induce adipogenesis [27]. BMP signaling exerts a context‐dependent dual role via the Smad and MAPK pathways [28], and the Wnt/β‐catenin pathway promotes bone formation while suppressing fat accumulation [27, 29]. The FGF/FGFR signaling pathway also functions as an essential molecular rheostat to sustain this balance, with FGF2 signaling promoting osteogenesis and restraining adipogenesis [9, 10]. However, excessive GC exposure severely disrupts this physiological balance, forcing BMSCs to undergo a pathological lineage shift that directly contributes to GIO [1, 30, 31]. Nonetheless, the precise molecular mechanisms mediating this fate determination remain to be fully elucidated. In this study, through unbiased RNA‐Seq analysis, we identified MACF1 as an essential regulator of this process. This regulatory role was further validated in vivo using a MACF1‐cKO mouse model, which exhibited severe bone loss accompanied by increased lipid droplet accumulation within the bone marrow cavity—a pathological hallmark strikingly similar to that of GIO, positioning MACF1 deficiency as a critical driver of the GC‐induced bone‐fat imbalance. The regulatory role of MACF1 in MSC fate determination was further confirmed in vitro, where MACF1 knockdown phenocopied the GC‐induced lineage shift, whereas its overexpression effectively corrected this fate bias. Furthermore, we developed a small activating RNA (saRNA) targeting the Macf1 promoter to upregulate its endogenous expression. Notably, saRNA‐mediated MACF1 activation not only restored osteogenic differentiation in GC‐treated MSCs but also rescued bone loss and improved bone microarchitecture in GIO mice. Collectively, these comprehensive in vivo and in vitro findings establish MACF1 as a crucial molecular switch in MSC fate determination, and underscore that its suppression is a prerequisite for the GC‐induced MSC fate bias and bone loss.

Small activating RNAs (saRNAs) are characterized as nucleic acid therapeutics that recruit transcriptional complexes to promoter regions to upregulate endogenous gene expression [32, 33, 34]. Utilizing this technology, which has demonstrated clinical efficacy in oncology [35, 36], we adopted a specific saRNA targeting the *Macf1* promoter as an interventional tool to counteract GC‐induced transcriptional suppression of endogenous MACF1. Following effective reactivation of endogenous MACF1, MSC osteogenic capacity was restored in vitro, and bone loss in GIO mice was significantly ameliorated in vivo. Although the rescue was incomplete, likely attributable to the broad‐spectrum inhibitory effects of GCs on additional osteogenic pathways such as Runx2 and Wnt signaling [1, 11, 31, 37], these findings validate MACF1‐targeted RNA activation as a novel, mechanism‐based therapeutic strategy for restoring bone homeostasis under long‐term GC exposure.

Beyond transcriptional reprogramming, MSC fate decisions rely on cytoskeletal‐driven intracellular trafficking to dictate the spatiotemporal distribution of key regulators [38, 39]. MACF1 is a crucial cytoskeletal cross‐linking protein that facilitates the connection between microtubules and actin filaments to regulate cellular functions [29, 44]. While our previous studies revealed that MACF1 promotes the osteogenic differentiation of BMSCs during aging through the HES1 or TCF4/miR‐335‐5p pathways [40, 41], its capacity to coordinate intracellular spatial trafficking has remained entirely unexplored. In this study, using Co‐IP coupled with LC‐MS/MS, we identified Rab14 as a novel MACF1‐binding partner. We further found that this binding was disrupted in GC‐treated and MACF1‐knockdown MSCs, whereas it was enhanced in MACF1‐overexpressing MSCs. In addition, Rab14 has been reported to recruit the motor protein KIF16B to drive FGFR vesicle trafficking [25, 42]. Therefore, we further examined the interaction between Rab14 and KIF16B, and the results showed that the Rab14/KIF16B complex was dismantled in both GC‐treated and MACF1‐knockdown MSCs, whereas its formation was significantly promoted in MACF1‐overexpressing cells. Crucially, these findings unveil a previously unrecognized biological function of MACF1, demonstrating that it acts as an indispensable molecular scaffold for Rab14/KIF16B complex assembly to orchestrate FGFR vesicular trafficking, thereby expanding its known roles into the realm of spatial receptor regulation.

The FGF/FGFR signaling axis is indispensable for MSC lineage commitment, playing a key role in maintaining the balance between osteogenesis and adipogenesis in MSCs [43, 44]. This signaling network comprises 22 structurally related FGF ligands and four highly conserved transmembrane tyrosine kinase receptors (FGFR1–4). Under physiological conditions, the binding of ligands to their cognate receptors triggers essential downstream cascades, such as the MAPK/Erk and PI3K/Akt pathways, which promote osteogenic programs while simultaneously suppressing adipogenic differentiation. Crucially, the activation of this molecular rheostat is contingent upon the spatiotemporal bioavailability of functional receptors at the plasma membrane [45, 46].

In BMSCs, FGFR1 and FGFR2 are established as abundantly expressed and functionally dominant receptor subtypes responsible for transducing these vital osteogenic signals [46, 47]. Furthermore, the canonical ligand FGF2 exerts its potent osteogenic effects predominantly by binding to and activating these two specific receptor subtypes [48, 49]. Consequently, in this study, we focused on tracking the subcellular distribution and vesicular transport of FGFR1 and FGFR2, alongside evaluating cellular sensitivity to exogenous FGF2. This targeted assessment provides a comprehensive representation of the dynamics of the FGF/FGFR axis. Following this rationale, our analysis revealed that the cell surface‐to‐total FGFR1/2 ratio was significantly decreased in both GC‐treated and MACF1‐knockdown MSCs, indicating an impairment in receptor trafficking to the plasma membrane. Interestingly, the increase in total FGFR1/2 levels observed in these deficient cells suggests a futile compensatory feedback loop, where synthesized receptors remain sequestered intracellularly due to a trafficking blockade rather than a synthesis failure. Conversely, MACF1 overexpression restored surface receptor density and rescued the attenuated osteogenic response to FGF2. Collectively, these results demonstrate that MACF1‐mediated trafficking efficiency is a prerequisite for ensuring FGF signaling, thereby safeguarding MSC fate against GC‐induced lineage bias without the metabolic burden of excessive, non‐functional receptor synthesis.

Furthermore, we interrogated public transcriptomic datasets of human bone samples to examine *MACF1* mRNA expression. In paired bone biopsies from patients with active Cushing's syndrome (GSE30159, a prototypical model for in vivo GC excess), MACF1 expression was suppressed, rebounding only after corrective treatment (Figure S9A). Consistent with these clinical findings, both mRNA and protein levels of MACF1 were decreased in GIO mice, suggesting that MACF1 downregulation represents a critical pathological event in GIO progression. Similarly, in hMSCs derived from primary osteoporosis patients (GSE35958), *MACF1* mRNA expression exhibited a downward trend compared with healthy controls, albeit with considerable individual heterogeneity. Additionally, we observed a significant upregulation of *FGFR1* and *FGFR2* mRNA levels in these osteoporosis patient samples (Figure S9B,C). This expression pattern was further validated in an independent hMSC dataset (GSE35956), which showed consistent downregulation of MACF1 and upregulation of FGFR1 and FGFR2 in osteoporosis patients compared with healthy controls (Figure S9D). Correspondingly, in our experimental systems, both GC treatment and MACF1 deficiency led to elevated FGFR1 and FGFR2 protein levels in MSCs, mirroring the dysregulation trend observed in the clinical datasets. These findings suggest that MACF1 deficiency disrupts FGFR homeostasis, whether through compensatory transcriptional activation or intracellular protein accumulation, and that this disruption serves as a critical pathological driver of osteogenic suppression.

To substantiate the trafficking‐dependent mechanism, we utilized the intracellular transport inhibitor BFA. BFA treatment recapitulated the key phenotypes induced by GC exposure, including reduced surface FGFR levels and suppressed osteogenic differentiation. Notably, while MACF1 overexpression effectively rescued the specific trafficking defects caused by GCs, it only partially ameliorated the broad‐spectrum upstream blockade imposed by BFA [50], confirming that MACF1 orchestrates downstream post‐Golgi delivery. Furthermore, Rab14 knockout provided genetic evidence that disruption of this trafficking axis concurrently impairs both osteogenic and adipogenic differentiation. Crucially, these Rab14‐deficient MSCs exhibited insensitivity to GC treatment, indicating that Rab14 functions as an obligate effector of GC action. Its disruption abolishes lineage competence and renders cells refractory to further pathogenic reprogramming.

Despite the comprehensive elucidation of the MACF1‐Rab14/KIF16B‐FGFR vesicular trafficking axis, a specific mechanistic limitation exists in the present study. The Act D assays demonstrated that *Macf1* mRNA stability was not altered by GCs (Figure S2G), indicating that MACF1 is more likely suppressed at the transcriptional level, consistent with the well‐established paradigm that GCs regulate gene expression through the GC/GR pathway rather than by inducing post‐translational protein degradation [51]. Nevertheless, the exact GC response elements on the MACF1 promoter and the specific corepressor complexes driving this suppression remain to be identified. In the future, advanced epigenetic mapping and promoter activity analyses will be employed to elucidate this upstream regulatory machinery.Furthermore, in this study, we have preliminarily elucidated that MACF1 regulates FGFR trafficking and BMSC fate by binding to Rab14 and recruiting KIF16B. However, the precise molecular mechanism underlying the interaction between MACF1 and the small GTPase Rab14 remains to be fully elucidated. Future structural biology studies, such as co‐crystallization or cryo‐electron microscopy analysis, may provide valuable insights into the precise molecular interface between MACF1 and Rab14, as well as the conformational changes that govern their GTP‐dependent interaction. Beyond this structural aspect, the detailed regulatory mechanism by which MACF1 orchestrates intracellular vesicular trafficking through Rab14 warrants further investigation.

## 4 Conclusion

In conclusion, this study establishes MACF1 as a director of FGFR vesicular trafficking, whose impairment acts as the driver of the pathogenic MSC fate shift in GIO. While the restoration of osteogenesis via targeted saRNA validates the reversibility of this defect, our findings reinforce the indispensable role of MACF1 in spatial receptor regulation. Consequently, the MACF1‐Rab14/KIF16B‐FGFR axis emerges as a critical biological target for restoring bone‐fat equilibrium in secondary osteoporosis.

## 5 Materials and Methods

### 5.1 Animal Models and Experimental Design

Male C57BL/6 mice (12 weeks old) were purchased from Beijing Vital River Laboratory Animal Technology Co., Ltd. (Beijing, China; License number: SCXK (Jing) 2021‐0011) and housed five per cage under controlled conditions (12‐h light/dark cycle, 24 ± 1°C). For GIO induction, mice received subcutaneous injections of Dex (Sigma–Aldrich, St. Louis, MO, USA, D4902) (low dose: 1 mg/kg; high dose: 5 mg/kg) or saline three times weekly for 8 weeks [52].

For saRNA treatment, mice were injected with Dex (2.5 mg/kg). One week post‐injection, mice were stratified into four groups: Blank, Mock, saNC, and saM. The saM (2.5 mg/kg) was complexed with the bone‐targeting RNA delivery system (SDSSD peptide‐modified polyvinylamine) at a 2:1 w/w ratio [53] and administered via tail vein injection weekly for 4 weeks. At the endpoint, mice were euthanized; femurs and tibias were harvested for micro‐CT scanning and histological analysis, respectively.

### 5.2 MACF1 Conditional Knockout (MACF1‐cKO) Mice Breeding

The generation strategy of MACF1‐cKO mice was described in detail in our previous study [41]. MACF1‐flox mice were generously provided by Associate Professor Xiaoyang Wu (University of Chicago, IL, USA). Prx1‐Cre mice were kindly provided by Professor Ge Zhang from Hong Kong Baptist University. Breeding of the MACF1‐cKO mouse model was performed as previously described [54, 55]. All mice were maintained and bred in a specific pathogen‐free facility, and animal experiments were conducted in accordance with institutional guidelines and with approval from the Committee of Animal Ethics and Experimental Safety.

### 5.3 Designation and Screening of MACF1 Promoter‐Targeted saRNA (saM)

Using miX‐RNA, a design software from Mixsungen, and based on published saRNA design guidelines [32, 56], four 19‐nt mouse *Macf1* promoter‐targeting saRNA double‐stranded oligonucleotides (mmu‐*Macf1*‐saM1 to saM4) were selected and synthesized in bulk. These saRNAs were designed to target the region spanning −1000 to −100 bp upstream of the *Macf1* transcription start site (TSS) annotated in the EPD database. The sequences of the saRNAs and saNC are listed in Table S5.

### 5.4 Primary Bone Marrow Mesenchymal Stem Cells (BMSCs) islation, Cell Culture and Transfection of saRNA

Primary BMSCs were isolated from male C57BL/6 mice (12 weeks old) or GIO mice. Following euthanasia, the femurs were promptly excised and stripped of surrounding soft tissues. The marrow cavity was repeatedly flushed with PBS via a 25‐gauge needle to harvest the marrow contents. This PBS‐marrow mixture underwent centrifugation at 1200 × g for 8 min. The resulting cell pellet was resuspended and mechanically dissociated using a 29‐gauge needle in complete growth medium, which consisted of Dulbecco's Modified Eagle Medium (Gibco, Grand Island, NY, USA, PM150210) enriched with 10% fetal bovine serum (FBS, Procell, Wuhan, Hubei, China, 164210), 1% L‐glutamine (Solarbio, Beijing, China, IG03902), and 1% penicillin‐streptomycin (Solarbio, Beijing, China, P1400). The cell suspension was then seeded into 60‐mm culture dishes and incubated at 37°C in a humidified 5% CO_2_ atmosphere for 3 h to allow initial cell adherence. Subsequently, non‐adherent cells were removed by gently washing with fresh medium. The remaining adherent BMSCs were maintained for an additional 36 h, with the medium refreshed at 12‐h intervals. Following this incubation, cells were subcultured into fresh plates to establish passage 1 (P1) cultures. All downstream phenotypic evaluations and experiments were conducted using cells at the third passage (P3).

Murine embryo fibroblast C3H10T1/2 cells were purchased from Xiamen Immocell Biotechnology Co., Ltd. (Xiamen, China, IM‐M057), and human mesenchymal stem cells (hMSCs) were purchased from Procell Life Science & Technology Co., Ltd. (Wuhan, China; CP‐H166). The cells were cultured in DMEM supplemented with 10% FBS, 1% L‐glutamine, and 1% penicillin‐streptomycin. The cells were maintained in a humidified 5% CO2 incubator at 37°C.

Primary BMSCs or C3H10T1/2 cells were seeded at a cell density of 2 × 10^4^ cells/cm^2^ and transfected with 50 nM *Macf1*‐saRNA (saM) or a negative control (saNC) using Lipofectamine Stem Transfection Reagent (Invitrogen, USA, STEM00003) according to the manufacturer's protocol. After a 6‐h transfection period, the medium was replaced with fresh complete growth medium. Cells were harvested at 72 h post‐transfection for qPCR analysis and at 96 h post‐transfection for Western blotting.

### 5.5 Osteogenic and Adipogenic Differentiation of the MSCs

Primary BMSCs were isolated from femurs as previously described, and C3H10T1/2 cells were cultured according to established protocols [41]. For differentiation assays, MSCs were cultured in osteogenic medium containing 10 mM β‐glycerophosphate (Solarbio, Beijing, China, G0210), 50 µg/mL ascorbic acid (Sigma‐Aldrich, St. Louis, MO, USA, A4403), and varying concentrations of Dex: 0.1 µM, 1 µM (low dose), and 5 µM (high dose). Osteoblast differentiation and matrix mineralization were analyzed using ALP staining and Alizarin Red S (ARS) staining as previously described. Similarly, adipogenic differentiation was induced using adipogenic induction medium containing 500 µM isobutylmethylxanthine (Solarbio, Beijing, China, II0010), 100 µM indomethacin (Solarbio, Beijing, China, II01001), and 10 µg/mL insulin (Solarbio, Beijing, China, II19102), alongside maintenance medium containing 10 µg/mL insulin and 5 µM pioglitazone (Solarbio, Beijing, China, IP02501). Both media were supplemented with corresponding concentrations of Dex (0.1, 1, or 5 µM). Lipid droplet accumulation was analyzed using Oil Red O staining as previously described.

### 5.6 Radiographic and Histologic Analyses

The trabecular morphometry of the mouse femurs was measured within the region 1 mm proximal to the distal growth plate using a Scanco µCT 40 microcomputed tomography system (Scanco Medical, Switzerland) at an 8‐µm isotropic resolution. For histological assessment, tibiae were fixed with 4% paraformaldehyde for 48 h, decalcified in EDTA for 4 weeks, embedded in paraffin, sectioned at a thickness of 5–10 µm, and stained with hematoxylin and eosin (H&E), or immunostained with specific antibodies against MACF1 (Abcam, Cambridge, MA, USA, ab117418), ALP (Santa Cruz Biotechnology, Dallas, TX, USA, sc‐365765), Runx2 (Santa Cruz Biotechnology, Dallas, TX, USA, sc‐390351), PPARγ (Santa Cruz Biotechnology, Dallas, TX, USA, sc‐7273), and FABP4 (Proteintech, Wuhan, Hubei, China, 12802‐1‐AP). For lipid droplet evaluation, the decalcified tibiae were cryosectioned and stained with BODIPY 493/503. The bone formation rate was determined by double calcein labeling. Mice were intraperitoneally injected with calcein (10 mg/kg, Solarbio, Beijing, China, C7600) 10 and 2 days before euthanasia. Undecalcified tibiae were fixed, embedded in plastic, and sectioned. Histological images were acquired using a fluorescence microscope (Nikon, Tokyo, Japan) and analyzed using ImageJ software (National Institutes of Health, Bethesda, MD, USA).

### 5.7 RNA‐Seq and Analysis

For mRNA‐seq analysis, raw data in FASTQ format were first processed using fastp (version 0.19.11) to remove low‐quality reads and adapters. The clean reads were then aligned to the reference genome using HISAT2 (or STAR, depending on your actual method). Subsequently, gene expression levels were quantified using featureCounts (subread package). Differential expression analysis was performed using the DESeq2 (or edgeR) R package. Genes with an adjusted *p*‐value < 0.05 and |log2(FoldChange)| > 1 were considered as differentially expressed genes (DEGs). Finally, KEGG pathway enrichment analysis of these DEGs was conducted using KOBAS software (or clusterProfiler).

### 5.8 Immunoprecipitation, Proteomic Analysis and Western Blot

Co‐IP assays were conducted using Protein A/G magnetic beads (MedChemExpress, Monmouth Junction, NJ, USA, gta) as previously described [26, 57]. For protein identification, immunoprecipitated complexes from C3H10T1/2 cells were resolved by SDS‐PAGE, and excised bands were digested and subjected to LC‐MS/MS analysis (Shanghai OE Biotech, Shanghai, China). The resulting LC‐MS/MS data were processed using Proteome Discoverer software (Thermo Fisher Scientific, Waltham, MA, USA) and systematically searched against the *Mus musculus* reference database to identify the captured proteins. Subsequently, relative protein quantification was performed based on normalized spectral abundance factors, and statistical significance between the target and IgG control groups was evaluated using Student's t‐tests with appropriate false discovery rate corrections. For interaction validation, lysates were immunoprecipitated with antibody‐bead complexes for 4 h at 4°C, followed by immunoblotting using antibodies against GAPDH (Proteintech, Wuhan, Hubei, China, 10494‐1‐AP), MACF1, Rab14 (Bioss, Beijing, China, bs‐2933R), KIF16B (Santa Cruz Biotechnology, Dallas, TX, USA, sc‐390309), and β‐tubulin (Proteintech, Wuhan, Hubei, China, 10068‐1‐AP), with total cell lysates serving as the input control.

For Western blot analysis, protein concentrations were determined using a BCA protein assay kit (Beyotime, Shanghai, China, P0009). Equal amounts of protein (20 µg/lane) were denatured at 105°C for 10 min, resolved by SDS‐PAGE, and electrophoretically transferred onto PVDF membranes (Merck Millipore, Burlington, MA, USA, IPVH00010). Following blocking with 5% non‐fat milk for 1 h, the membranes were probed with specific primary antibodies overnight at 4°C. After extensive washing with TBST, the membranes were incubated with HRP‐conjugated secondary antibodies for 1 h at room temperature. Protein bands were subsequently visualized using a chemiluminescence imaging system. The specific primary antibodies used in this study included: anti‐Erk1/2 (Proteintech, Wuhan, Hubei, China, 11257‐1‐AP), anti‐phospho‐Erk1/2 (Thr202/Tyr204) (Proteintech, Wuhan, Hubei, China, 80031‐1‐RR), anti‐β‐tubulin (Proteintech, Wuhan, Hubei, China, 10068‐1‐AP), and anti‐GAPDH (Proteintech, Wuhan, Hubei, China, 10494‐1‐AP). HRP‐conjugated anti‐rabbit (Proteintech, Wuhan, Hubei, China, SA00001‐2) or anti‐mouse IgGs (Proteintech, Wuhan, Hubei, China, SA00001‐1) were used as secondary antibodies.

### 5.9 Vesicle Immune‐isolation and SEM

MSCs were homogenized in cold lysis buffer via a 27‐gauge needle. The post‐nuclear supernatant (500 × g) was pre‐cleared with agarose beads and subjected to immunoprecipitation using an anti‐MACF1 antibody and Protein A/G magnetic beads (MedChemExpress, Monmouth Junction, NJ, USA, HY‐K0202). To preserve vesicle structure, all bead separation steps were performed via magnetic separation rather than centrifugation. The isolated bead‐bound vesicles were washed, resuspended, and visualized using a scanning electron microscope (SEM, HITACHI, Tokyo, Japan, SU6600).

### 5.10 RNA Extraction and q‐PCR Analysis

Total RNA from osteoblasts and bone tissues was extracted using TRIzol reagent (Life Technologies, Carlsbad, CA, USA, 15596018). Reverse transcription was performed using the PrimeScript RT reagent Kit (TaKaRa, Kusatsu, Shiga, Japan, RR036A) according to the manufacturer's instructions. Quantitative real‐time PCR (qPCR) was carried out using ChamQ SYBR qPCR Master Mix (Vazyme, Nanjing, Jiangsu, China, Q311). Relative mRNA levels were calculated using the 2^−ΔΔCT^ method and normalized to *Gapdh* expression. The specific primer sequences used in this study are listed in Table S6.

### 5.11 Plasmid Construction

Full‐length cDNAs encoding mouse *Fgfr1*, *Kif16b*, and *Rab14* were amplified by PCR from mouse BMSC‐derived cDNA using KOD‐Plus DNA Polymerase (Toyobo, Osaka, Japan, KOD‐201) and the specific primers listed in Table S7. The resulting amplicons were then ligated into pEGFP‐C1 or pmCherry plasmids to construct the mCherry‐FGFR1, mCherry‐Rab14, and EGFP‐KIF16B expression vectors using standard cloning methods.

### 5.12 Time Lapse Observation

For time‐lapse confocal microscopy, MSCs were cultured on gelatin‐coated Lab‐Tek 35‐mm chambered coverslips (Nunc, Rochester, NY, USA) in DMEM supplemented with 10% FBS and 1% penicillin‐streptomycin. The cells were maintained at 37°C in a humidified 5% CO2 atmosphere overnight. Transfection with the expression vectors was performed using Lipofectamine Stem Transfection Reagent (STEM00015) and Opti‐MEM (51‐985‐034) (both from Invitrogen, Carlsbad, CA, USA) according to the manufacturer's protocol. Sixteen hours post‐transfection, live cells were analyzed using a TCS SP5 confocal laser scanning microscope (Leica Microsystems, Wetzlar, Germany). The acquired images were processed and contrast‐enhanced using ImageJ software (National Institutes of Health, Bethesda, MD, USA).

### 5.13 Cytosolic and Membrane Fractionation

Membrane and cytosolic fractions were isolated using a Membrane and Cytosol Protein Extraction Kit (Beyotime, Shanghai, China, P0033) following the manufacturer's protocol. Briefly, cells were homogenized in Reagent A supplemented with PMSF. After removing nuclei and cellular debris via centrifugation at 700 × g for 10 min, the supernatant was centrifuged at 14 000 × g for 30 min at 4°C. The resulting supernatant was collected as the cytosolic fraction, while the pellet was resuspended in Reagent B to obtain the membrane fraction. Protein concentrations were quantified using a BCA protein assay kit.

### 5.14 Study Approval

All animals were housed in the animal facility of the China Astronaut Research and Training Center (Beijing, China). All experimental procedures were approved by the Laboratory Animal Administration Committee of Xi'an Medical University and conducted in strict accordance with the institutional Guidelines for Animal Experimentation and the Guide for the Care and Use of Laboratory Animals (NIH Publication No. 85‐23, revised 2011).

### 5.15 Statistical Analysis

All statistical analyses were performed using GraphPad Prism 8.4.3 (GraphPad Software, La Jolla, CA, USA). Data are presented as the mean ± standard deviation (SD) from at least three independent biological replicates. Technical replicates were defined as independent parallel measurements acquired from the same biological specimen. Prior to parametric testing, data normality and homogeneity of variance were systematically verified using the Shapiro‐Wilk and Brown‐Forsythe tests, respectively. For pairwise comparisons, unpaired two‐tailed Student's t‐tests were utilized. For comparisons involving more than two groups, one‐way or two‐way analysis of variance (ANOVA) followed by Tukey's post hoc test was applied to account for multiple comparisons. Statistical significance was set at *p* < 0.05.

## Author Contributions

All authors reviewed the manuscript. P.S., Y.T., and F.Z.: Writing – review & editing, Writing – original draft, Investigation, Funding acquisition. J.L., S.P., Y.T., J.W., and J.A.L.: Writing – original draft, Investigation. C.Z.: Data mining and analysis of human samples. A.X. and L.Z.: Writing – review & editing, Investigation. H.G.: Funding acquisition. X.C. and A.Q.: Funding acquisition, Validation. Q.Y.: Funding acquisition, Supervision, Project administration.

## Funding

This work was supported by the National Natural Science Foundation of China (Grant Nos. 82204543, 32371296, 32371371, 82400996, and 81900399), the Natural Science Foundation of Guangdong Province (Grant No. 2024A1515013078), the Innovation Capability Support Program of Shaanxi (Grant No. 2025RS‐CXTD‐056), and the Key Laboratory Project of the Shaanxi Provincial Department of Education (Grant No. 23JS053)

## Conflicts of Interest

The authors declare no conflicts of interest.

## Supporting information




**Supporting File 1**: advs77073‐sup‐0001‐SuppMat.docx.

**Supporting File 2**: advs77073‐sup‐0002‐TableS1.xlsx.

**Supporting File 3**: advs77073‐sup‐0003‐TableS2.xlsx.

**Supporting File 4**: advs77073‐sup‐0004‐TableS3.xlsx.

**Supporting File 5**: advs77073‐sup‐0005‐TableS4.xlsx

**Supporting File 6**: advs77073‐sup‐0006‐TableS1.mp4.

**Supporting File 7**: advs77073‐sup‐0006‐TableS3.mp4.

**Supporting File 8**: advs77073‐sup‐0007‐TableS2.mp4.

**Supporting File 9**: advs77073‐sup‐0007‐TableS4.mp4.

**Supporting File 10**: advs77073‐sup‐0008‐TableS5.mp4.

**Supporting File 11**: advs77073‐sup‐0009‐TableS6.mp4.

## Data Availability

The datasets used and/or analyzed during the current study are available from the corresponding author on reasonable request.
