# Supplementary material for: Dysregulation of the MACF1‐Rab14/KIF16B‐FGFR Vesicular Trafficking Axis Skews MSC Lineage Commitment in Glucocorticoid‐Induced Osteoporosis
Source: Adv Sci (Weinh). 2026 Aug 10:e77073. Online ahead of print. doi: 10.1002/advs.77073 (PMC13455833; doi:10.1002/advs.77073)
Supplement: Supplementary file 1 [file ADVS-9999-e77073-s010.docx]

**Table S1. List of proteins identified by LC-MS/MS in sh-NC cells following immunoprecipitation with IgG control antibody.**

This table provides a comprehensive list of proteins co-immunoprecipitated with a nonspecific IgG antibody from lysates of sh-NC (control) C3H10T1/2 cells. Protein identifications were performed using liquid chromatography-tandem mass spectrometry (LC-MS/MS). This dataset serves as a negative control to establish baseline nonspecific binding for the MACF1-specific immunoprecipitations presented in Table S2. The table includes protein accessions, gene names, peptide counts, and sequence coverage where applicable.

**Table S2. List of MACF1-interacting proteins identified by LC-MS/MS in sh-NC cells following immunoprecipitation with MACF1 antibody.**

This table represents the baseline MACF1 interactome in sh-NC cells, serving as the reference for comparison with MACF1-deficient conditions (Table S4).

**Table S3. List of proteins identified by LC-MS/MS in sh-MACF1 cells following IgG control immunoprecipitation.**
This table serves as a negative control for nonspecific binding in MACF1-knockdown (sh-MACF1) cells, complementing the IgG control in sh-NC cells (Table S1).

**Table S4. List of MACF1-interacting proteins identified by LC-MS/MS in sh-MACF1 cells following MACF1 immunoprecipitation.**
This table represents the MACF1 interactome remaining after MACF1 knockdown, allowing identification of MACF1-specific interactors by comparison with Table S2.

**Table S5. The sequences of saRNA**

| **The saRNA** | **Sequence** |
| --- | --- |
| saM1 sense strand | 5’-CUACUGAUUGUCAGGCAGAAA-3’ |
| saM1 antisense strand | 5’-UCUGCCUGACAAUCAGUAGUA-3’ |
| saM2 sense strand | 5’-UCCUUGUAGGAGACCUGACUU-3’ |
| saM2 antisense strand | 5’-GUCAGGUCUCCUACAAGGAUA-3’ |
| saM3 sense strand | 5’-GUCACCCUCGACAUUCUCACC-3’ |
| saM3 antisense strand | 5’-UGAGAAUGUCGAGGGUGACAA-3’ |
| saM4 sense strand | 5’-UAGCUAGGCCUGCCUGGUAGA-3’ |
| saM4 antisense strand | 5’-UACCAGGCAGGCCUAGCUAAA-3’ |
| saNC sense strand | 5’-UUCUCCGAACGUGUCACGUTT-3’ |
| saNC antisense strand | 5’-ACGUGACACGUUCGGAGAATT-3’ |

**Table S6. The sequences of primers for RT-PCR used in this study**

| **Primer** | **Sequence** |
| --- | --- |
| *Pparγ* forward | 5’-CCAAGAATACCAAAGTGCGATCA-3’ |
| *Pparγ* reverse | 5’-CCCACAGACTCGGCACTCAAT-3’ |
| *Fabp4* forward | 5’-AAGAAGTGGGAGTGGGCTTTG-3’ |
| *Fabp4* reverse | 5’-CTCTTCACCTTCCTGTCGTCTG-3’ |
| *Alp* forward | 5’-GTTGCCAAGCTGGGAAGAACAC-3’ |
| *Alp* reverse | 5’-CCCACCCCGCTATTCCAAAC-3’ |
| *Runx2* forward | 5’-CGCCCCTCCCTGAACTCT-3 |
| *Runx2* reverse | 5’-TGCCTGCCTGGGATCTGTA-3’ |
| *Col I* forward | 5’-GAAGGCAACAGTCGATTCACC-3’ |
| *Col I* reverse | 5’-GACTGTCTTGCCCCAAGTTCC-3’ |
| *Macf1* forward | 5’-GAAAACATTCACCAAGTGGGTCAAC-3’ |
| *Macf1* reverse | 5’-TGTCCATCCCGAAGGTCTTCATAG-3’ |
| *Abca1* forward | 5’-GGGCTCCTCCCTGTTTTTGA-3’ |
| *Abca1* reverse | 5’-TCTGAGAAACACTGTCCTCCTTTT-3’ |
| *β-actin* forward | 5’-GCACCGTCAAGGCTGAGAAC-3’ |
| *β-actin* reverse | 5’-AGCACTGTGTTGGCGTACAG-3’ |

**Table S7. The sequences of primers for plasmid construction used in this study**

| **Primer** | **Sequence** |
| --- | --- |
| *Fgfr1* forward | 5’-GGGGGGAAGCTTGCCACCATGGTCAGCTGGGGGCGCTTCA-3’ |
| *Fgfr1* reverse | 5’-CGCGGTACCATGCGCCGTTTGAGTCCACTGTTGG-3’ |
| *Rab14* forward | 5’-GCTTCCATGGCAACTGCACCGTACAACTACT-3 |
| *Rab14* reverse | 5’-GTACCCTAGCAGCCACAGCCTTCTCTCTGGG-3’ |
| *Kif16b* forward | 5’-GCAGTCGACATGGCATCGGTCAAGGTGG-3’ |
| *Kif16b* reverse | 5’-CCGCGGTACCCTACCCTGTCCCATGGCTG-3’ |
